# Influenza and Pneumococcal Vaccination in Non-Infected Cardiometabolic Patients from the Americas during the COVID-19 Pandemic. A Sub-Analysis of the CorCOVID-LATAM Study

**DOI:** 10.3390/vaccines9020123

**Published:** 2021-02-04

**Authors:** Álvaro Sosa Liprandi, Ezequiel José Zaidel, Ricardo Lopez Santi, John Jairo Araujo, Manuel Alfonso Baños González, Juan Martín Busso, Luz Cabral, Jorge Camilletti, Juan Erriest, Roberto Flores, Ezequiel Forte, Mirecly Guzman Ramos, Máxima Mendez Castillo, Leonardo Josué Ramírez Zambrano, Carmen Roa, Piero Custodio–Sanchez, Gustavo Solache Ortiz, Bernardo Spitz, Adrián Baranchuk

**Affiliations:** 1 Sanatorio Güemes, Buenos Aires C1180AAX, Argentina; ezaidel@fsg.edu.ar; 2 Hospital Italiano de La Plata, La Plata B1900, Argentina; lopezsan@live.com.ar (R.L.S.); jcamil@speedy.com.ar (J.C.); erriestjuan@gmail.com (J.E.); 3 Centro Cardiovascular Somer Incare, Rionegro 054040, Colombia; Johnjairoaraujo@gmail.com; 4 Cardiology Department, Universidad Juarez Autónoma de Tabasco, Villahermosa Tabasco 86040, Mexico; manuel_banos@hotmail.com; 5 Sanatorio San José, Buenos Aires c1425, Argentina; j.m.busso@gmail.com; 6 Centro Médico Nacional-Hospital Nacional Itauguá, Itauguá 2740, Paraguay; luzcabral@yahoo.com; 7 Hospital Ramón Carrillo, Santiago del Estero G4200, Argentina; roberto_flores77@yahoo.es; 8 CENDIC Centro Diagnóstico Cardiovascular, Concordia E3202, Argentina; ezeforte@yahoo.com; 9 IVSS Dr Luis Guada Lacau, Valencia 2300, Venezuela; mireclyguzman@gmail.com; 10 CEDIMAT, Santo Domingo 10514, Dominican Republic; drammendez18@gmail.com; 11 Centro Clínico San Cristobal, San Cristobal 5001, Venezuela; cardiolecturas@gmail.com; 12 Hospital Metropolitano de Santiago, Santo Domingo 51000, Dominican Republic; encarnacionroa@yahoo.es; 13 Hospital Nacional Almanzor Aguinaga Asenjo -Essalud, Chiclayo 14001, Peru; custodiomed@hotmail.com; 14 Instituto de Cardiología Preventiva, San Juan del Río 76800, Mexico; gustavosolache@gmail.com; 15 Clínica Cuyo, Mendoza M5500, Argentina; spitzbeni@gmail.com; 16 Queen’s University, Kingston, ON K7L 3N6, Canada; Adrian.Baranchuk@kingstonhsc.ca

**Keywords:** COVID-19, SARS-CoV-2, Influenza vaccination, pneumococcal vaccination, cardio–metabolic

## Abstract

Background: Influenza vaccination (IV) and Pneumococcus vaccination (PV) are recommended for patients with cardiometabolic diseases. This study aimed to evaluate the immunization rate of ambulatory cardiometabolic patients during the COVID-19 pandemic in the Americas. Methods: Electronic surveys were collected from 13 Spanish speaking countries between 15 June and 15 July 2020. Results: 4216 patients were analyzed. Mean age 60 (±15) years and 49% females. Global IV rate was 46.5% and PV 24.6%. Vaccinated patients were older (IV = 63 vs. 58 years; PV = 68 vs. 59, *p* < 0.01) but without gender difference. Vaccination rates were greater in higher-risk groups (65+, diabetics, heart failure), but not in coronary artery disease patients. In the Southern cone, the rate of IV and PV was approximately double that in the tropical regions of the Americas. In a multivariate model, geographic zone (IV = OR 2.02, PV = OR 2.42, *p* < 0.001), age (IV = OR 1.023, PV = OR 1.035, *p* < 0.001), and incomes (IV = OR 1.28, PV = OR 1.58, *p* < 0.001) were predictors for vaccination. Conclusions: During the COVID-19 pandemic, ambulatory patients with cardiometabolic diseases from the Americas with no evidence of COVID-19 infection had lower-than-expected rates of IV and PV. Geographic, social, and cultural differences were found, and they should be explored in depth.

## 1. Introduction

There is a strong relation between respiratory infections and acute cardiovascular (CV) events. Any Influenza strains and also Streptococcus pneumoniae infections can trigger a variety of CV alterations that may lead to hospitalization or even death [1,2,3,4,5].

The body of evidence confirms that Influenza vaccination (IV) and Pneumococcal vaccination (PV) are related to a decrease in the rate of different CV outcomes, such as myocardial infarction (MI), heart failure (HF) hospitalization, and CV mortality [6,7,8,9,10,11,12,13,14,15,16]. However, some barriers were identified for implementing these prevention strategies, which are grouped in patient’s decision (vaccine hesitancy, prior experience, fake news), health care providers knowledge and attitude towards vaccination, and health care system barriers, that lead to a lower than expected immunization rate in Latin America and globally [17,18,19,20,21,22,23,24,25]. In that struggling context, at the end of 2019, a new coronavirus SARS-CoV-2 provoked a pandemic particularly prevalent in the Americas. In North America, it coincided with the end of winter, while in South America, it rose in June and July 2020 [26].

Governmental authorities and health leaders from most countries recommended, on the one hand, to avoid the circulation of people on the streets and in-home confinement, but on the other hand, suggested that vaccination against respiratory pathogens should be kept as usual, and that may have been perceived as a contradictory message. Currently, the information regarding immunization rates for both primary and secondary cardiometabolic disease prevention in the Americas is unknown.

This study aimed to describe the rate of immunization with IV and PV in ambulatory cardiometabolic patients from the Americas with no evidence of infection during the COVID-19 pandemic and to analyze their determinants.

## 2. Materials and Methods

A pre-specified analysis of the CorCOVID LATAM survey was performed. The rationale and design of the main study have been previously published [27]. It was a survey sponsored by the InterAmerican Society of Cardiology (SIAC) and performed by cardiologists from 13 Spanish-speaking countries to patients older than 18 years with prior CV or metabolic diseases, including arterial hypertension, coronary artery disease, cardiomyopathies, heart failure, valvular disease, pericardial disease, obesity, dyslipidemia, or diabetes, with no evidence or history of COVID-19 infection, with the aim to assess the impact of the COVID-19 pandemic on habits, lifestyle, access to healthcare and treatments, as well as psychological factors [28]. The information was gathered in an online platform (Google Forms platform, Mountain View, CA) between 15 June and 15 July 2020.

Specific inclusion criteria were all subjects at cardiology consultation with cardiovascular or metabolic disease and without evidence or suspicion of being sick from SARS-CoV2. Exclusion criteria were patients less than 18 years old, a previous diagnosis of SARS-CoV2, recent hospital stay, or not willing to provide the information contained in the survey. The questionnaire was divided into seven clusters, including basic demographic data and specific vaccine information. A total of 38 questions were performed on each patient. Informed consent was obtained from all the participants in accordance with the requirements of the InterAmerican Society of Cardiology Ethics Committee. Due to government measures to limit population mobilization in some countries, the survey was conducted either by face-to-face visits or by phone or video chat, in which case, informed consent was verbally taken. The basic demographic profile included educational level (illiterate, elementary, high school, tertiary, or university), income (very low, low, middle, and high), occupation, family structure and habitat, cardiovascular disease, and treatments. The second part of the survey examined the patient’s behavior during the last 30 days regarding physical activity, nutrition, body weight, alcohol intake and tobacco habits, treatments’ accessibility, mood, and quality of sleep. The questions contained multiple answer options and were not forced so that patients were allowed to select multiple responses depending on the question.

The main objective of this sub study was to describe the Influenza and Pneumococcal vaccination rates in cardiometabolic patients from Spanish speaking countries of Latin America. The secondary goals were (1) to assess predictors of IV and PV, (2) to describe the immunization rate in high-risk groups, (3) to analyze geographical, economic, and cultural differences regarding IV and PV within Latin America, (4) to describe predictors of immunization among diabetes, heart failure, and coronary artery disease patients.

The responses of the included patients were classified according to the country of origin in three regions: Mexico, Central America, and the Caribbean (Costa Rica, Cuba, El Salvador, Guatemala, Mexico, Dominican Republic), Andean region (Colombia, Ecuador, Peru, Venezuela), and Southern cone (Argentina, Chile, Paraguay).

IV was considered positive if one dose of any type of IV was received in the last 6 months, while for PV, the time frame was 3 years for at least one dose (without considering different types of PVs). Patients who had received both IV and PV were analyzed in the “double vaccination” group.

IV and PV comparative analyses were performed, stratified by age, sex, and region of inclusion. Specific group aims were elder (65 years or more), patients with known coronary artery disease (CAD), patients with heart failure (HF), and patients with diabetes without prior CV events. In addition, determinants of immunization, such as educational and socioeconomic level, were evaluated in univariate and adjusted models.

Statistical analysis: dichotomic variables are described as numbers and their percentages, and continuous variables are described as mean (±standard deviation) or median (interquartile range) in accordance with their distribution type. χ^2^ and Fisher’s tests were used for comparisons between categorical variables, while for comparisons between continuous variables, the Student’s *t*-test or Mann–Whitney U test was used. Bartlett’s test was used to determine the normality of the data distribution of the continuous variables. To assess independent predictors of vaccination, multivariate models were performed in the full cohort and stratified by specific at-risk groups (coronary artery disease, heart failure, and diabetes in primary prevention). In the absence of data on any variable, no imputation was made. The Epi Info^®^V7.0 program was used, and a *p*-value of < 0.05 was considered significant. For the multivariate models, variables with a *p* < 0.1 on univariate analysis were included. Bonferroni correction was used because of the exploratory nature of the analysis and multiple comparisons [29], considering *p* = 0.00625 for the Influenza vaccine full cohort analysis (8 variables model), and 0.00714 for the Pneumococcal vaccine model (7 variables). Further analyses of predictors of IV, PV, and double vaccination in specific risk-groups of CAD patients, HF patients, and diabetic patients were performed with multivariable models. In them, a *p*-value of 0.0083 was considered significant.

For this study, the Strengthening the Reporting of Observational studies in Epidemiology (STROBE) recommendations [30] were followed.

## 3. Results

From 4429 surveys, 213 had to be excluded due to incomplete data, inclusion criteria violation, or duplicates. Thus, 4216 remained for analyses. The mean age was 60 (±15) years, and 49% were females. Immunization variables were complete in all cases. One thousand, seven hundred and sixty-four (42%) patients were older than 65 years, 899 (21.3%) were diabetics, and 606 (14.3%) were smokers (or former smokers). Seven hundred and sixty-nine (18.2%) patients had known CAD, and 538 (12.7%) had HF. The global IV rate was 46.5% (n = 1963), PV 24.6% (n = 1039), and double vaccination 21% (n = 887). Baseline characteristics are shown in Table 1. A homogeneous distribution in the three geographic areas was achieved: 1426 patients were from Mexico and Central America, 1359 from the Andean region, and 1431 from the Southern cone.

We found no gender difference in vaccination uptake (Influenza vaccination: 46.8% in females and 46.4% in males, OR = 1.01, CI 95% 0.89–1.14, *p* = 0.8; Pneumococcus vaccination: 23.9% in females vs. 25.4% in males, OR = 0.92, CI 95% 0.80–1.06, *p* = 0.25). Vaccinated patients were older for IV (63 (±15) vs. 58 (±14) years, *p* < 0.001), PV (68 (IQR 58–75) vs. 59 (IQR 48–70) years, *p* < 0.001), and double vaccination (69 (70–76) vs. 59 (49–70) years, *p* < 0.001). Table 2 shows that vaccination rates were greater in specific high risk groups, such as elder (65+), diabetics, and HF patients. However, the vaccination rate was similar in CAD in comparison with non-CAD patients. Current smokers or past smokers had a lower immunization rate for Influenza, Pneumococcus, and double vaccination.

**Figure 1:**
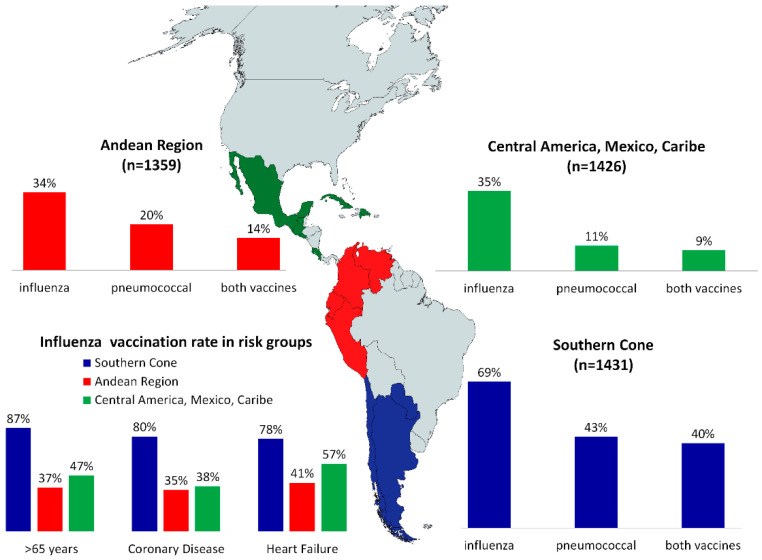
Immunization rate by geographic zone.

On exploratory analyses, differences in immunization rates between geographic regions, educational level, and economic strata were analyzed. Patients from the Southern cone had vaccination rates of approximately double than the tropical regions (Figure 1): Influenza vaccination 69% in Southern cone, 34% in Andean region, and 35% in Mexico, Central America and Caribbean (χ^2^ = 452, df = 2, *p* < 0.001); Pneumococcus vaccination 43%, 20%, and 11%, respectively (χ^2^ = 406, df = 2, *p* < 0.001); and double vaccination 40%, 11%, and 9%, respectively (χ^2^ = 458, df = 2, *p* < 0.001).

The specific countries with higher immunization rates were Argentina (65%), Peru (54%), and Chile (36%) for PV, and for IV: Chile (91%), Argentina (87%), and Paraguay (70%). Patients from the Southern cone within the high-risk population (65+, CAD, HF) had the highest Influenza immunization rates (87%, 80%, and 78%, respectively), and it doubled the rate to other regions (Figure 1, all *p* < 0.001)

**Figure 2:**
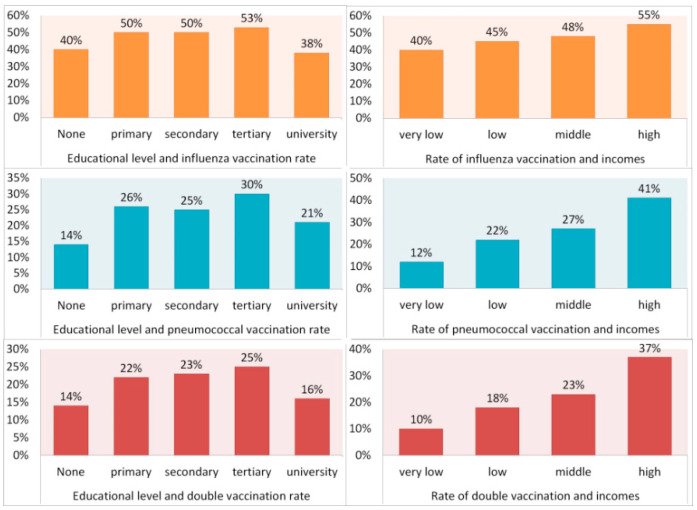
Relation between educational and economic levels and immunization rates.

We found an incremental correlation between economic strata and vaccines uptake in this cohort, in spite being free of charge in most countries (Figure 2): for IV, 40%, 45%, 48%, and 55%, in very low, low, middle, and high incomes, respectively (χ^2^ = 18.9, df = 3, *p* < 0.001). For PV, 12%, 22%, 27%, 41%, (χ^2^ = 84.1, df = 3, *p* < 0.001), and for double vaccination the rates were 10%, 18%, 23%, and 37%, respectively (χ^2^ = 77.3, df = 3, *p* < 0.001). On the other hand, we found significant differences regarding educational level (IV, χ^2^= 61.9, df = 4, *p* < 0.001), (PV, χ^2^ = 29.4.9, df = 4, *p* < 0.001) (double vaccination, χ^2^ = 33.4, df = 4, *p* < 0.001), but it did not follow an incremental correlation, lowering the immunization rates among mostly educated patients (Figure 2). The rate of IV was 40% in illiterate patients and 38% in university level patients.

In a multivariate analysis, after adjustment for the factors found in the bivariate model and the exploratory socio-demographic analysis, the stronger predictors of vaccines rates in all groups were the geographic zone (Southern cone having the highest odds), age (approximately 3% increase per aging year), and income level (Table 3), both for IV and PV. Heart failure was associated with IV only, and diabetes was not associated with IV or PV after Bonferroni’s adjusted *p*-value. Smoking was an independent predictor of non-vaccination for IV and PV.

An analysis of specific risk groups was performed. Among CAD patients (n = 769), geographic zone, age, and incomes level were the stronger predictors for PV and for IV (OR = 2.3289, 95% CI = 1.913–2.8351; OR = 1.0385, 95% CI = 1.0236–1.0535; OR = 1.6188, 95% CI = 1.2643–2.0727, respectively, for IV),

(OR = 1.9265, 95% CI= 3.1583–5.4228; OR = 1.034, 95% CI = 1.016−1.0523; OR = 1.5372, 95% CI = 1.1392–2.0743, respectively, for PV). In patients with diabetes but without HF or CAD (n = 460), geographic zone, and age were independent predictors of IV and PV. (OR = 1.9265, 95% CI = 1.5077–2.4617; and OR = 1.0266, 95% CI = 1.0103–1.0432, respectively, for IV) (OR = 2.4263, 95% CI = 1.8359–3.2065; and OR = 1.0348, 95% CI = 1.0155–1.0545, respectively, for PV). However, for HF patients (n = 538) independent predictors were the same for IV but for PV the predictors were geographic zone, income level, and educational level (OR = 1.8039, 95% CI = 1.3591–2.3944; OR = 1.0189, 95% CI = 1.0044–1.0336; and OR = 1.5697, 95% CI = 1.1688−2.1079, respectively, for IV) (OR = 2.6097, 95% CI = 1.9048–3.5755; OR = 1.5093, 95% CI = 1.1014–2.0684, and OR = 1.3809, 95% CI = 1.1026–1.7294, respectively, for PV).

## 4. Discussion

The main findings of this study were that during the COVID-19 pandemic, immunization against Influenza was low and against Pneumococcus was very low in ambulatory cardiometabolic patients from the Americas, and there were significant socio-cultural and geographic differences in vaccination rates among them. The risk group with a higher immunization rate was the elder group (65+ years). That may be related to the wider knowledge from doctors and patients regarding the general benefits of immunization in this group, opposite to immunization for younger patients with HF, diabetes, or CAD. As mentioned before, there is a strong correlation between respiratory infections and CV events, as well as a clear cut down with vaccination, and currently, vaccines are recommended in several cardiology guidelines [31,32,33,34,35]. It is possible that the COVID-19 pandemic had an impact on these low rates, something that needs further exploration.

Immunization rates were higher in the Southern cone zone compared to the other regions. Knowledge about respiratory viruses’ circulation, the attack rate of Influenza, and seasonality may be lower by doctors and patients from tropical countries, and then immunization may not be taken into account as a CV prevention strategy among them. Regarding differences in immunization rates among regions and countries analyzed, IV is recognized in the essential medicines list (EML) only in 4 out of 13 countries (Argentina, Dominican Republic, El Salvador, Mexico), and PV in 8 countries (Argentina, Colombia, Dominican Republic, Ecuador, El Salvador, Mexico, Paraguay, and Peru). Being listed in the EML is the first step towards improvement in availability and access.

Due to the nature of this survey, a transversal cut trial during the COVID-19 pandemic, we compared the immunization rates with the Pan American Health Organization (PAHO) reported rates of immunization in high-risk groups from prior years, and noted that, as an example, IV rates were near 100% in several regions of the Americas for 65+ group in the pre-pandemic years [36], and thus, we assume that we are probably facing a strong decrease in immunization rates against respiratory pathogens this season, with potential adverse consequences on public health for further months or years, indirectly related to the COVID-19 pandemic. Other preventive health measures, such as cancer screening, also had modifications during the pandemic, with unknown later outcomes [37,38,39,40,41,42].

By the time WHO declared the pandemic, several restrictive measures were taken, including the suspension of non-urgent medical care and CV disease care. As an example, in prior publications, we noted more than a 70% reduction in elective CV procedures during March and April of 2020 (compared to an average from 2010–2019) in Buenos Aires, as well as a fall in hospitalizations for acute CV events, such as coronary syndromes or HF hospitalizations [43]. In Latin America, the low rate of availability and use of telehealth and digital prescriptions before the COVID-19 pandemic, together with the lack of access to clinics and cardiologic follow-up (related to each country’s restrictive governmental measures), may have contributed, at least in part, to lower access to vaccines.

Pneumococcal vaccination rates were very low in all high-risk groups. Pneumococcus and PV do not follow a seasonal pattern, but IV campaigns are an opportunity for PV uptake. Fifty-one percent of the included patients had a prior CV hospitalization, and half of them had a hospitalization in 2019–2020, making a stronger case for the PV prescription during the analyzed time frame and possibly reflecting a low prescription or acceptance by patients of PV during the pandemic. Although some patients may have received the complete PV scheme before the last three years, these results are alarming. In the surveys from the tropical countries, the rate was as low as 10%. The later development and knowledge about CV benefits of PV in comparison to IV [44] recall bias, direct cost, and lack of coverage in some national immunization programs, as well as its availability and distribution issues during the COVID-19 pandemic, may have been determinants of this low rate.

Heart failure is a usual indication for Influenza and Pneumococcus immunization, irrespective of the age of the patients, who usually live with dyspnea and are at higher risk of pneumonia and developing in-hospital complications [14,15,16]. Our data showed that HF was indeed a predictor of the use of vaccines. However, the proportional weight was milder than socio-demographic predictors than income level, age, or geographic area. Although the number of HF patients in this survey was low, this may reflect that in the HF population, age surpasses HF itself as the main reason for immunization. Of note, in the largest cohorts, HF average age was around 70 years [45,46].

Several ecologic and molecular studies found that patients with coronary artery disease are vulnerable and at risk of respiratory infections [1,2,3,4]. Then, randomized clinical trials, retrospective studies, and metanalyses have shown that immunization reduces mortality and morbidity among them [11,12,13,47,48,49,50]. However, in this cohort, known CAD was not associated with increased rates of immunization. Knowledge, attitude, and behavior both by patients and physicians may play a role in this finding, as well as psychological aspects of CAD patients after an acute coronary event [51].

Opposite to previously described predictors of IV and PV, smoking status had a strong, independent, and inverse relationship with vaccination. Even under cardiac care, smokers are a known group of non-adherence to preventive health measures. The risk of severe pneumonia, as well as the benefit of vaccination, is neglected by them [52,53,54,55,56].

Beyond direct pandemic effects regarding immunization rates, this study revealed inequities in the access to IV and PV as preventive CV measures in the Americas. Low income patients and low educational level patients had significantly lower rates of vaccination in spite of vaccines being incorporated in most national immunization programs free of charge. Knowledge, information in mass media, access to consultation and prescriptions, should be reviewed as barriers in achieving greater vaccination rates in this vulnerable cohort. During the COVID-19 pandemic, this inequity in access to healthcare was more visible, according to several studies [57,58,59,60,61]. Even more, global gross domestic product shrinkage of 3.2% in 2020 was predicted by the United Nations, and that will push 34 million more people into extreme poverty [62] with a potentially worse scenario for IV and PV rates for the next years if proper measures are not taken by stakeholders.

On the other edge, the immunization rate for both IV and PV declined in the stratum of the higher educational level (university level). This phenomenon may be attributed to chance and the low number of surveys, but unfortunately, we found similar reports regarding lower vaccination levels in the most educated participants [63], attributed to skeptical views and sentiments related to vaccine safety and efficacy. Of note, that study also included patients from the Americas. The low immunization rate, even in the context of a pandemic caused by a new respiratory virus, is a call to action for the development of strategies to increase the rate of Influenza and Pneumococcal vaccination for the vulnerable population of the Americas.

This survey was performed at the half of 2020 when COVID-19 was increasing in the Americas, and information regarding circulating Influenza was limited due to redirection of resources to the care of the pandemics, and epidemiological surveillance for other viruses was halted. Currently, we know that the Influenza rate during 2020 was 0.2% in Southern Cone, 4.4% in the Andean region, 2.7% in Central America, 4.8% in the Caribbean region, and 6.4% in North America (PAHO report) [64]. Influenza A H1N1pdm09 and B Yamagata were the predominant strains during 2020. By week 51, there were 28,281 CRP confirmed H1N1pdm09 cases, 112,415 Influenza B cases, and 139,655 Influenza A not subtyped in the Americas, but at the same time, there were 13,351,225 SARS-CoV-2 confirmed cases. The uptake of SARS-CoV-2, social distancing, and the use of masks may have contributed to this phenomenon, being the lowest Influenza season in decades. There is uncertainty regarding the uptake of Influenza once SARS-CoV-2 diminishes (naturally or due to vaccination) or when social distancing measures reduce.

Finally, the world is in need of rapid development and access to COVID-19 vaccines. In the race, mRNA-based and adenovirus-based vaccines have been in use since December 2020. The long-term duration of immunity of the vaccines or the diseases themselves, as well as developing mutations of the virus, are not well documented so far. Thus, there is a potential need for repeated SARS-CoV-2 vaccination, and in that context, prior experience of IV campaigns and fusion of efforts towards an improvement in adult vaccinations may contribute to global health.

### Limitations

For IV, the 6 months window analyzed included the usual Influenza season and the IV campaign months for Southern Cone, Andean countries, and some Central American countries. However, it is possible that for Mexico and the Dominican Republic, the period analyzed captured only the last part of the winter and IV prescription, which may have biased the results of part of Central America, Mexico, and the Caribbean region.

It is possible that patients who agreed to participate and are under cardiac follow-up are more adherent to health care strategies, including immunization, compared to the general population. Despite this, immunization levels were lower than in the years before the pandemic and much lower than recommended levels. Regarding predictors of immunization in the specific subgroup analyses (patients with CAD, with HF, or with diabetes), geographic zone and age were again independent predictors of IV and PV in all, and income level in some subgroups. In spite of being similar to the findings from the whole cohort multivariable analyses, these results should be taken with caution, as the number of cases included for each specific analysis was low.

## 5. Conclusions

During the COVID-19 pandemic, ambulatory patients with cardiometabolic diseases from the Americas had lower-than-expected rates of Influenza and Pneumococcal vaccination, both in primary and secondary prevention of CV disease. Geographic, social, and cultural differences were found, and they should be explored in depth.

## Figures and Tables

**Table 1:** Clinical characteristics of the population.

Age	60.35 (SD 15.39)
Female sex	49.07% (2069)
Region	
-Mexico, Central America	33.82% (1426)
-Andean	32.23% (1359)
-Southern cone	33.94% (1431)
Educational Level	
-None (analphabet)	2.28% (96)
-Primary	19.14% (807)
-Secondary (high school)	32.14% (1355)
-Tertiary	15.32% (646)
-University	31.12% (1312)
Incomes	
-Very low (needs external support)	10.01% (422)
-Low (covers basic needs)	35.53% (1498)
-Middle (can save)	48.55% (2047)
-High	5.91% (249)
Coronary artery disease	18.24% (769)
Diabetes	21.32% (899)
Hypertension	72.84% (3071)
Heart failure	12.70% (538)
Current or past smokers	14.3% (616)
Increased smoking during pandemic	17.00% (103)
Influenza vaccination in the last 6 months	46.56% (1963)
Pneumococcal vaccination in the last 3 years	24.64% (1039)

**Table 2:** Rate of immunization with Influenza vaccine, Pneumococcal vaccine, and double vaccination in specific risk groups.

		Influenza Vaccination				Pneumococcal Vaccination				Both Vaccines			
		Yes (*n* = 1963)	No (*n* = 2253)	OR (CI)	*p*	Yes (*n* = 1963)	No (*n* = 3177)	OR (CI)	*p*	Yes (*n* = 887)	No (*n* = 3329)	OR (CI)	*p*
**Age > 65y**	Yes (*n* = 1764)	1048 (59%)	716 (41%)	2.45 (2.16–2.77)	<0.001	624 (35%)	1140 (65%)	2.68 (2.32–3.1)	<0.001	565 (32%)	1199 (68%)	3.12 (2.67–3.64)	<0.001
	No (*n* = 2442)	913 (37%)	1529 (63%)			413 (16%)	2029 (64%)			320 (13%)	2122 (87%)		
**T2DM**	Yes (*n* = 899)	456 (51%)	443 (49%)	1.23 (1.06–1.43)	0.002	245 (27%)	654 (73%)	1.19 (1.01–1.41)	0.023	216 (24%)	683 (76%)	1.24 (1.04–1.48)	0.008
	No (*n* = 3317)	1507 (45%)	1810 (55%)			794 (24%)	2523 (76%)			671 (20%)	2646 (80%)		
**Smoking**	Yes (*n* = 616)	237 (39%)	369 (61%)	0.7 (0.59–0.83)	<0.001	77 (13%)	529 (87%)	0.43 (0.31–0.51)	<0.001	65 (11%)	541 (89%)	0.41 (0.31–0.53)	<0.001
	No (*n* = 3610)	1726 (48%)	1884 (52%)			962 (27%)	2648 (73%)			822 (23%)	2788 (77%)		
**Heart** **Failure**	Yes (*n* = 538)	302 (56%)	236 (44%)	1.55 (1.29–1.86)	<0.001	160 (30%)	378 (70%)	1.34 (1.1–1.64)	0.002	142 (26%)	396 (74%)	1.41 (1.14–1.73)	<0.001
	No (*n* = 3687)	1661 (45%)	2017 (55%)			879 (24%)	2799 (76%)			745 (20%)	2933 (80%)		
**Coronary** **Disease**	Yes (*n* = 769)	381 (50%)	388 (50%)	1.15 (0.99–1.35)	0.06	182 (24%)	587 (76%)	0.93 (0.78–1.12)	0.48	159 (21%)	610 (79%)	0.97 (0.8–1.18)	0.78
	No (*n* = 3477)	1582 (46%)	1865 (54%)			857 (25%)	2590 (75%)			728 (21%)	2719 (79%)		

**Table 3:** Multivariate analysis, independent predictors of Influenza vaccination and Pneumococcal vaccination.

Independent Predictors of Influenza Vaccination, Full Cohort (n = 4216)						
Term	Odds Ratio	95% C.I.	Coefficient	S. E.	Z-Statistic	*p*-Value
Age (per year)	1.023	1.018–1.028	0.023	0.002	9.558	**0.0000**
Heart failure	1.360	1.097–1.686	0.308	0.109	2.81	**0.0049**
Diabetes	1.214	1.037–1.423	0.194	0.080	2.410	0.0159
Coronary artery disease	0.993	0.836–1.180	−0.006	0.087	−0.070	0.9441
Smoking	0.762	0.632–0.919	−0.271	0.095	−2.846	**0.0044**
Educational level	0.989	0.925–1.058	−0.010	0.034	−0.304	0.761
Income level	1.283	1.162–1.415	0.249	0.050	4.966	**0.0000**
Geographic Zone	2.012	1.854–2.184	0.699	0.041	16.721	**0.0000**
**Independent Predictors of Pneumococcal Vaccination, Full Cohort (n = 4216)**						
**Term**	**Odds Ratio**	**95% C.I.**	**Coefficient**	**S. E.**	**Z-Statistic**	***p*-Value**
Age (per year)	1.035	1.029–1.041	0.035	0.003	11.709	**0.0000**
Heart Failure	1.386	1.087–1.767	0.326	0.123	2.637	0.0084
Diabetes	1.203	1.003–1.446	0.184	0.094	1.963	0.0496
Smoking	0.451	0.346–0.587	−0.796	0.134	−5.194	**0.0000**
Educational level	1.077	0.994–1.167	0.074	0.040	1.832	0.0668
Income level	1.582	1.405–1.782	0.459	0.060	7.566	**0.0000**
Geographic Zone	2.472	2.234–2.737	0.905	0.051	17.483	**0.0000**

Note: With Bonferroni correction, a *p* level of 0.00625 was considered for the Influenza vaccine full cohort analysis (8 variables model), and 0.00714 for the Pneumococcal vaccine model (7 variables). Statistical significative results are shown in bold.

## Data Availability

The data presented in this study are available on request from the corresponding author.

## References

[1] Warren-Gash C., Smeeth L., Hayward A.C. (2009). Influenza as a trigger for acute myocardial infarction or death from cardiovascular disease: A systematic review. Lancet. Infect. Dis..

[2] Clayton T.C., Thompson M., Meade T.W. (2008). Recent respiratory infection and risk of cardiovascular disease: Case-control study through a general practice database. Eur. Heart J..

[3] Kwok C.S., Aslam S., Kontopantelis E., Myint P.K., Zaman M.J., Buchan I., Loke Y.K., Mamas M.A. (2015). Influenza, influenza-like symptoms and their association with cardiovascular risks: A systematic review and meta-analysis of observational studies. Int. J. Clin. Pract..

[4] Madjid M., Miller C.C., Zarubaev V.V., Marinich I.G., Kiselev O.I., Lobzin Y.V., Filippov A.E., Casscells S.W. (2007). Influenza epidemics and acute respiratory disease activity are associated with a surge in autopsy-confirmed coronary heart disease death: Results from 8 years of autopsies in 34,892 subjects. Eur. Heart J..

[5] Kwong J.C., Schwartz K.L., Campitelli M.A., Chung H., Crowcroft N.S., Karnauchow T., Katz K., Ko D.T., McGeer A.J., McNally D. (2018). Acute Myocardial Infarction after Laboratory-Confirmed Influenza Infection. N. Engl. J. Med..

[6] Hung I.F., Leung A.Y., Chu D.W., Leung D., Cheung T., Chan C.K., Lam C.L., Liu S.H., Chu C.M., Ho P.L. (2010). Prevention of acute myocardial infarction and stroke among elderly persons by dual pneumococcal and influenza vaccination: A prospective cohort study. Clin. Infect. Dis. Off. Publ. Infect. Dis. Soc. Am..

[7] Nichol K.L. (2005). Influenza vaccination in the elderly: Impact on hospitalisation and mortality. Drugs Aging.

[8] Nichol K.L., Margolis K.L., Wuorenma J., Von Sternberg T. (1994). The efficacy and cost effectiveness of vaccination against influenza among elderly persons living in the community. N. Engl. J. Med..

[9] Nichol K.L., Nordin J., Mullooly J., Lask R., Fillbrandt K., Iwane M. (2003). Influenza vaccination and reduction in hospitalizations for cardiac disease and stroke among the elderly. N. Engl. J. Med..

[10] Bridges C.B., Fukuda K., Uyeki T.M., Cox N.J., Singleton J.A., Centers for Disease Control and Prevention, Advisory Committee on Immunization Practices (2002). Prevention and control of influenza: Recommendations of the Advisory Committee on Immunization Practices (ACIP). Mmwr. Recomm. Rep. Morb. Mortal. Wkly. Rep. Recomm. Rep..

[11] Udell J.A., Zawi R., Bhatt D.L., Keshtkar-Jahromi M., Gaughran F., Phrommintikul A., Ciszewski A., Vakili H., Hoffman E.B., Farkouh M.E. (2013). Association between influenza vaccination and cardiovascular outcomes in high-risk patients: A meta-analysis. JAMA.

[12] Clar C., Oseni Z., Flowers N., Keshtkar-Jahromi M., Rees K. (2015). Influenza vaccines for preventing cardiovascular disease. Cochrane Database Syst. Rev..

[13] LeBras M.H., Barry A.R. (2017). Influenza Vaccination for Secondary Prevention of Cardiovascular Events: A Systematic Review. Can. J. Hosp. Pharm..

[14] Lamontagne F., Garant M.P., Carvalho J.C., Lanthier L., Smieja M., Pilon D. (2008). Pneumococcal vaccination and risk of myocardial infarction. CMAJ Can. Med. Assoc. J..

[15] Vila-Corcoles A., Ochoa-Gondar O., Rodriguez-Blanco T., Gutierrez-Perez A., Vila-Rovira A., Gomez F., Raga X., de Diego C., Satue E., Salsench E. (2012). Clinical effectiveness of pneumococcal vaccination against acute myocardial infarction and stroke in people over 60 years: The CAPAMIS study, one-year follow-up. BMC Public Health.

[16] Rodrigues B.S., David C., Costa J., Ferreira J.J., Pinto F.J., Caldeira D. (2020). Influenza vaccination in patients with heart failure: A systematic review and meta-analysis of observational studies. Heart.

[17] Davis B.M., Black D. (2017). Identifying the Challenges to Adult Influenza Vaccination in Latin America. Value Health.

[18] Zimmerman R.K., Santibanez T.A., Fine M.J., Janosky J.E., Nowalk M.P., Bardella I.J., Raymund M., Wilson S.A. (2003). Barriers and facilitators of pneumococcal vaccination among the elderly. Vaccine.

[19] (1999). Centers for Disease Control and Prevention (CDC) Reasons reported by Medicare beneficiaries for not receiving influenza and pneumococcal vaccinations--United States, 1996. Mmwr. Morb. Mortal. Wkly. Rep..

[20] Centers for Disease Control (1988). Adult immunization: Knowledge, attitudes, and practices—DeKalb and Fulton Counties, Georgia, 1988. JAMA.

[21] Bovier P.A., Chamot E., Bouvier Gallacchi M., Loutan L. (2001). Importance of patients’ perceptions and general practitioners’ recommendations in understanding missed opportunities for immunisations in Swiss adults. Vaccine.

[22] Schmid P., Rauber D., Betsch C., Lidolt G., Denker M.L. (2017). Barriers of Influenza Vaccination Intention and Behavior—A Systematic Review of Influenza Vaccine Hesitancy, 2005–2016. PLoS ONE.

[23] Burki T.K. (2019). Vaccine misinformation and social media. Lancet Digit. Health.

[24] Hill J.A., Agewall S., Baranchuk A., Booz G.W., Borer J.S., Camici P.G., Chen P.S., Dominiczak A.F., Erol Ç., Grines C.L. (2019). Medical Misinformation: Vet the Message!. Int. J. Cardiol..

[25] Chan M.S., Jamieson K.H., Albarracin D. (2020). Prospective associations of regional social media messages with attitudes and actual vaccination: A big data and survey study of the influenza vaccine in the United States. Vaccine.

[26] PAHO COVID Status in the Americas. https://www.paho.org/es/temas/coronavirus/brote-enfermedad-por-coronavirus-covid-19.

[27] Lopez Santi R., Piskorz D.L., Marquez M.F., Ramirez Ramos C., Renna N.F., Ibarrola M., Wyss F.S., Naranjo Dominguez A., Perez G.E., Farina J.M. (2020). Impact of the Pandemic on NonInfected Cardiometabolic Patients: A Survey in Countries of Latin America-Rationale and Design of the CorCOVID LATAM Study. CJC Open.

[28] Piskorz D., Barragán A.P., Lopez Santi R. (2020). Psychological impact of the pandemic on ambulatory cardiometabolic patients without evidence of SARS-CoV-2 infection. The CorCOVIDLatamPsy Study. Curr Probl Cardiol..

[29] Armstrong R.A. (2014). When to use the Bonferroni correction. Ophthalmic Physiol. Opt..

[30] Von Elm E., Altman D.G., Egger M., Pocock S.J., Gøtzsche P.C., Vandenbroucke J.P., STROBE Initiative (2007). The Strengthening the Reporting of Observational Studies in Epidemiology (STROBE) statement: Guidelines for reporting observational studies. Lancet.

[31] Ponikowski P., Voors A.A., Anker S.D., Bueno H., Cleland J.G., Coats A.J., Falk V., González-Juanatey J.R., Harjola V.P., Jankowska E.A. (2016). The Task Force for the diagnosis and treatment of acute and chronic heart failure of the European Society of Cardiology (ESC). Developed with the special contribution of the Heart Failure Association (HFA) of the ESC. Eur. J. Heart Fail..

[32] Knuuti J., Wijns W., Saraste A., Capodanno D., Barbato E., Funck-Brentano C., Prescott E., Storey R.F., Deaton C., Cuisset T. (2020). ESC Scientific Document Group 2019 ESC Guidelines for the diagnosis and management of chronic coronary syndromes. Eur. Heart J..

[33] Amsterdam E.A., Wenger N.K., Brindis R.G., Casey D.E., Ganiats T.G., Holmes D.R., Jaffe A.S., Jneid H., Kelly R.F., Kontos M.C. (2014). 2014 AHA/ACC Guideline for the Management of Patients with Non-ST-Elevation Acute Coronary Syndromes: A report of the American College of Cardiology/American Heart Association Task Force on Practice Guidelines. J. Am. Coll. Cardiol..

[34] Yancy C.W., Jessup M., Bozkurt B., Butler J., Casey D.E., Drazner M.H., Fonarow G.C., Geraci S.A., Horwich T., Januzzi J.L. (2013). American Heart Association Task Force on Practice Guidelines 2013 ACCF/AHA guideline for the management of heart failure: A report of the American College of Cardiology Foundation/American Heart Association Task Force on Practice Guidelines. J. Am. Coll. Cardiol..

[35] MacIntyre C.R., Mahimbo A., Moa A.M., Barnes M. (2016). Influenza vaccine as a coronary intervention for prevention of myocardial infarction. Heart.

[36] PAHO Influenza Vaccination in the Americas by Risk Group. https://www.paho.org/en/topics/influenza.

[37] Mitchell E.P. (2020). Declines in Cancer Screening During COVID-19 Pandemic. J. Natl. Med. Assoc..

[38] Khargekar N.C., Khanna D. (2020). Cancer Screening during COVID-19 Pandemic. Asia-Pac. J. Oncol. Nurs..

[39] Mazzone P.J., Gould M.K., Arenberg D.A., Chen A.C., Choi H.K., Detterbeck F.C., Farjah F., Fong K.M., Iaccarino J.M., Janes S.M. (2020). Management of Lung Nodules and Lung Cancer Screening During the COVID-19 Pandemic: CHEST Expert Panel Report. Chest.

[40] Shaukat A., Church T. (2020). Colorectal cancer screening in the USA in the wake of COVID-19. Lancet Gastroenterol. Hepatol..

[41] Curigliano G., Cardoso M.J., Poortmans P., Gentilini O., Pravettoni G., Mazzocco K., Houssami N., Pagani O., Senkus E., Cardoso F. (2020). Recommendations for triage, prioritization and treatment of breast cancer patients during the COVID-19 pandemic. Breast.

[42] Vose J.M. (2020). Delay in Cancer Screening and Diagnosis During the COVID-19 Pandemic: What Is the Cost?. Oncology.

[43] Vensentini N., Zaidel E.J., Charask A., Salzberg S., Gagliardi J., Perea J., Sosa Liprandi Á., de Abreu M., Mariani J., Tajer C.D. (2020). Internaciones cardiovasculares en Unidades de Cuidados Intensivos durante la pandemia por COVID-19 [Cardiovascular admissions in Intensive Care Units during COVID-19 pandemic]. Medicina.

[44] Christenson B., Hedlund J., Lundbergh P., Ortqvist A. (2004). Additive preventive effect of influenza and pneumococcal vaccines in elderly persons. Eur. Respir. J..

[45] Bui A.L., Horwich T.B., Fonarow G.C. (2011). Epidemiology and risk profile of heart failure. Nat. Rev. Cardiol..

[46] Conrad N., Judge A., Canoy D., Tran J., Pinho-Gomes A.C., Millett E., Salimi-Khorshidi G., Cleland J.G., McMurray J., Rahimi K. (2019). Temporal Trends and Patterns in Mortality after Incident Heart Failure: A Longitudinal Analysis of 86,000 Individuals. JAMA Cardiol..

[47] Gurfinkel E.P., de la Fuente R.L., Mendiz O., Mautner B. (2002). Influenza vaccine pilot study in acute coronary syndromes and planned percutaneous coronary interventions: The FLU Vaccination Acute Coronary Syndromes (FLUVACS) Study. Circulation.

[48] Ciszewski A., Bilinska Z.T., Brydak L.B., Kepka C., Kruk M., Romanowska M., Ksiezycka E., Przyluski J., Piotrowski W., Maczynska R. (2008). Influenza vaccination in secondary prevention from coronary ischaemic events in coronary artery disease: FLUCAD study. Eur. Heart J..

[49] Phrommintikul A., Kuanprasert S., Wongcharoen W., Kanjanavanit R., Chaiwarith R., Sukonthasarn A. (2011). Influenza vaccination reduces cardiovascular events in patients with acute coronary syndrome. Eur. Heart J..

[50] Keshtkar-Jahromi M., Vakili H., Rahnavardi M. (2009). The efficacy of influenza vaccination in reducing cardiovascular events in patients with coronary artery diseases: IVCAD study. Clin. Microbiol. Infect..

[51] Lane D., Carroll D., Ring C., Beevers D.G., Lip G.Y. (2002). The prevalence and persistence of depression and anxiety following myocardial infarction. Br. J. Health Psychol..

[52] Looijmans-van den Akker I., van den Heuvel P.M., Verheij T.J., van Delden J.J., van Essen G.A., Hak E. (2007). No intention to comply with influenza and pneumococcal vaccination: Behavioural determinants among smokers and non-smokers. Prev. Med..

[53] Nuorti J.P., Butler J.C., Farley M.M., Harrison L.H., McGeer A., Kolczak M.S., Breiman R.F. (2000). Cigarette smoking and invasive pneumococcal disease. Active Bacterial Core Surveillance Team. N. Engl. J. Med..

[54] Whitney C.G., Schaffner W., Butler J.C. (2001). Rethinking recommendations for use of pneumococcal vaccines in adults. Clin. Infect. Dis..

[55] Godoy P., Castilla J., Soldevila N., Mayoral J.M., Toledo D., Martín V., Astray J., Egurrola M., Morales-Suarez-Varela M., Domínguez A. (2018). Smoking may increase the risk of influenza hospitalization and reduce influenza vaccine effectiveness in the elderly. Eur. J. Public Health.

[56] Wong C.M., Yang L., Chan K.P., Chan W.M., Song L., Lai H.K., Thach T.Q., Ho L.M., Chan K.H., Lam T.H. (2013). Cigarette smoking as a risk factor for influenza-associated mortality: Evidence from an elderly cohort. Influenza Other Respir. Viruses.

[57] Germain S., Yong A. (2020). COVID-19 Highlighting Inequalities in Access to Healthcare in England: A Case Study of Ethnic Minority and Migrant Women. Fem. Leg. Stud..

[58] Bambra C., Riordan R., Ford J., Matthews F. (2020). The COVID-19 pandemic and health inequalities. J. Epidemiol. Community Health.

[59] O’Dowd A. (2020). Covid-19 pandemic is magnifying healthcare inequalities, says England’s regulator. BMJ Clin. Res..

[60] Grohskopf L.A., Liburd L.C., Redfield R.R. (2020). Addressing Influenza Vaccination Disparities During the COVID-19 Pandemic. JAMA.

[61] McLaughlin J.M., Swerdlow D.L., Khan F., Will O., Curry A., Snow V., Isturiz R.E., Jodar L. (2019). Disparities in uptake of 13-valent pneumococcal conjugate vaccine among older adults in the United States. Hum. Vaccines Immunother..

[62] United Nations (2020). World Economic Situation and Prospects as of mid-2020. https://www.un.org/development/desa/dpad/wp-content/uploads/sites/45/publication/WESP2020_MYU_Report.pdf.

[63] Larson H.J., de Figueiredo A., Xiahong Z., Schulz W.S., Verger P., Johnston I.G., Cook A.R., Jones N.S. (2016). The State of Vaccine Confidence 2016: Global Insights Through a 67-Country Survey. EBioMedicine.

[64] PAHO Influenza Weekly Report. https://www.paho.org/es/documentos/actualizacion-regional-influenza-semana-epidemiologica-50-22-diciembre-2020.

